# Blended Smoking Cessation Treatment: Exploring Measurement, Levels, and Predictors of Adherence

**DOI:** 10.2196/jmir.9969

**Published:** 2018-08-01

**Authors:** Lutz Siemer, Marjolein GJ Brusse-Keizer, Marloes G Postel, Somaya Ben Allouch, Angelos Patrinopoulos Bougioukas, Robbert Sanderman, Marcel E Pieterse

**Affiliations:** ^1^ Research Group Technology, Health & Care Saxion University of Applied Sciences Enschede Netherlands; ^2^ Centre for eHealth and Well-being Research University of Twente Enschede Netherlands; ^3^ Medical School Twente Medisch Spectrum Twente Enschede Netherlands; ^4^ Tactus Addiction Treatment Enschede Netherlands; ^5^ Department of Health Psychology University Medical Center Groningen University of Groningen Groningen Netherlands

**Keywords:** blended treatment, smoking, adherence, predictors, tobacco, prevention, cognitive behavioral therapy

## Abstract

**Background:**

Blended face-to-face and Web-based treatment is a promising way to deliver cognitive behavioral therapy. Since adherence has been shown to be a measure for treatment’s acceptability and a determinant for treatment’s effectiveness, in this study, we explored adherence to a new blended smoking cessation treatment (BSCT).

**Objective:**

The objective of our study was to (1) develop an adequate method to measure adherence to BSCT; (2) define an adequate degree of adherence to be used as a threshold for being adherent; (3) estimate adherence to BSCT; and (4) explore the possible predictors of adherence to BSCT.

**Methods:**

The data of patients (N=75) were analyzed to trace adherence to BSCT delivered at an outpatient smoking cessation clinic. In total, 18 patient activities (eg, using a Web-based smoking diary tool or responding to counselors’ messages) were selected to measure adherence; the degree of adherence per patient was compared with quitting success. The minimum degree of adherence of patients who reported abstinence was examined to define a threshold for the detection of adherent patients. The number of adherent patients was calculated for each of the 18 selected activities; the degree of adherence over the course of the treatment was displayed; and the number of patients who were adherent was analyzed. The relationship between adherence and 33 person-, smoking-, and health-related characteristics was examined.

**Results:**

The method for measuring adherence was found to be adequate as adherence to BSCT correlated with self-reported abstinence (*P*=.03). Patients reporting abstinence adhered to at least 61% of BSCT. Adherence declined over the course of the treatment; the percentage of adherent patients per treatment activity ranged from 82% at the start of the treatment to 11%-19% at the final-third of BSCT; applying a 61% threshold, 18% of the patients were classified as adherent. Marital status and social modeling were the best independent predictors of adherence. Patients having a partner had 11-times higher odds of being adherent (OR [odds ratio]=11.3; CI: 1.33-98.99; *P*=.03). For social modeling, graded from 0 (=partner and friends are not smoking) to 8 (=both partner and nearly all friends are smoking), each unit increase was associated with 28% lower odds of being adherent (OR=0.72; CI: 0.55-0.94; *P*=.02).

**Conclusions:**

The current study is the first to explore adherence to a blended face-to-face and Web-based treatment (BSCT) based on a substantial group of patients. It revealed a rather low adherence rate to BSCT. The method for measuring adherence to BSCT could be considered adequate because the expected dose-response relationship between adherence and quitting could be verified. Furthermore, this study revealed that marital status and social modeling were independent predictors of adherence.

**Trial Registration:**

Netherlands Trial Registry NTR5113; http://www.trialregister.nl/trialreg/admin/rctview.asp?TC=5113 (Archived by WebCite at http://www.webcitation.org/71BAPwER8).

## Introduction

### Smoking Cessation Treatment

As smoking is the leading cause of preventable death, cessation treatment remains pivotal for public health promotion. In past decades, a variety of effective interventions for smoking cessation have become available [1,2], including, more recently, Web-based interventions [3,4] and mobile-phone interventions [5,6]. Currently, both traditional and Web-based modes of delivery are being increasingly merged into blended treatment.

### Blended Treatment

Blended treatment is a promising way to deliver behavioral change interventions as it allows combining the strengths of face-to-face treatment (personal attention of a professional, allowing for rich and dynamic synchronous communication) with the unique features of Web-based care (accessibility anytime and anywhere, self-paced asynchronous communication) [7-12]. In the recent past, a growing body of research on blended treatment has emerged [9,13] exploring diverse aspects such as individual and group treatments [14] for a number of health issues, such as depression [15], anxiety [16], and addiction [11]; comparing modes of delivery, such as mainly Web-based [17,18], mainly face-to-face [16,19], and 50-50 [20]; orders of modes of delivery, such as integrated [11] and sequential [18]; and tools used, such as platforms, emails, short message service text messaging, and apps [21,22].

### Adherence

While blended treatment may decrease dropout rates [9], it may also increase adherence, which is often low in both Web-based and cessation treatments [23]. Adherence can be defined as the extent to which a person’s behavior—taking medication, following a diet, or executing lifestyle changes—corresponds with recommendations from a health care provider [24]. In the context of behavioral change treatments (eg, smoking cessation counseling), issues of adherence are mostly related to premature termination of the treatment and failures to complete between-session tasks and exercises [25]. Low adherence is both an indicator for limited treatment acceptability and a primary determinant of treatment effectiveness [24,26-28] because it leads to suboptimal exposure of patients to evidence-based components of treatment, which in turn—assuming a dose-response relationship—negatively affects treatment outcome [24].

### Adherence to Blended Treatment

Until now, little has been known about adherence to blended treatment. In a randomized controlled trial (RCT; N=97), comparing the blended treatment of comorbid mental health and substance use problems with face-to-face treatment, participants were found to be equally able to engage, bond, and commit to treatment [29]. However, in another RCT (N=45), adherence was significantly lower for blended depression treatment than for face-to-face treatment (90.5% vs 95.1%), although both treatments were equally effective [30]. Based on a small sample (N=9) in another blended depression treatment trial, adherence rates were considered promising (ie, 5 of 7 patients who started blended treatment completed 90% of it) [20]. This initial evaluation study also revealed that discontinuing blended treatment appeared to be unrelated to the blended nature of the treatment and, unsurprisingly, having internet access and a functional computer at home was indispensable. Finally, a case report on blended treatment for antepartum depression also showed good adherence [31].

In the context of smoking cessation, to the best of our knowledge, adherence to blended treatment has not been assessed. For smoking cessation treatment in general, adherence rates widely vary between studies (5%-96%), which can be explained by differences in the interventions used, adjunctive support, and populations studied [24]. Typically, in a smoking cessation treatment, adherence rapidly declines over the initial weeks of treatment, followed by a more gradual decrease in the later stages, resulting in rather low adherence rates (<40%) [24].

### Predictors of Adherence

As adherence is pivotal for treatment effectiveness [24], predicting adherence becomes relevant because it may increase treatment efficacy. Adherence, in general, is determined by provider behaviors, health system factors, and personal characteristics [24]. In particular, the latter have been examined as predictors of adherence to traditional interventions [32]. However, similar studies on adherence to blended treatment appear to be lacking. Within the context of smoking cessation treatment—including both face-to-face and Web-based treatments—several person-, smoking-, and health-related predictors of adherence have been examined. The likelihood of being adherent increases with a higher age [33,34], male gender [34], higher internet skills [35,36], negative attitude toward smoking and higher motivation to quit at baseline [37,38], higher self-efficacy at baseline [38], early success in quitting after the start of the treatment [26,33,39], and lower nicotine dependency at baseline and fewer withdrawal symptoms after quitting [34,37]. The question arises whether these predictors apply to blended treatment as well.

### Measurement of Adherence

To examine the predictors of adherence, valid measurement of adherence becomes a prerequisite. Taking into account both the novelty and diversity of blended treatments, one can understand that established measures for adherence to blended treatment in particular are still lacking. Therefore, for the purpose of this study, a customized measure was constructed based on a combination of parameters used for face-to-face and Web-based interventions. In face-to-face treatment, adherence is often operationalized as completion of tasks assigned during the treatment or the number of completed or attended treatment sessions [40]. In Web-based treatment, the measures of adherence often comprise log-ins to programs, module completion, time spent online, (self-reported) completion of predefined activities such as use of an Web-based tool, posts made, pages viewed, replies to emails, forum visits, or print requests made [23]. Aiming to increase precision and accuracy, the adherence measure developed for this study was primarily based on objective or direct adherence indicators, such as whether or not a patient attended a face-to-face session, responded to a counselor’s message, or used a certain Web-based treatment tool (eg, “goal setting” or “think differently”). Using observable and digitally traceable patient activities, limitations in terms of reliability and validity of self-report data [41] can be largely avoided.

### Thresholds Defining “Adequate” and “Inadequate” Adherence

Finally, in addition to measuring adherence as a continuous variable, applying a categorical measure based on a threshold for “adequate” and “inadequate” adherence may be useful for clinical purposes [32]. However, justifications for the operationalizations of thresholds are a common issue; a recent review [42] on adherence to eHealth revealed that 28 of 62 studies described thresholds, but only 6 reported a justification for the threshold. In line with Carolan [43], in this study, we have defined the threshold in relation to the treatment outcome (ie, quitting smoking). To the best of our knowledge, neither for blended smoking cessation treatment (BSCT), in particular, nor for blended treatment, in general, have the thresholds for adherence been explored until now. In the context of smoking cessation, intervention thresholds for classifying participants as adherent or nonadherent range from 75% to 100% use of intervention components offered [24].

### Objectives

In view of all that has been mentioned so far, the objectives of this exploratory study were as follows:

1. To develop a method to measure adherence to a BSCT by selecting traceable activities of the patients and to determine whether this method is adequate by comparing the degree of adherence with the quitting success (ie, verifying the expected dose-response relationship between adherence and quitting).
2. To define an adequate degree of adherence to be used as a threshold to detect adherent patients by examining the minimum degree of adherence of the patients who reported abstinence.
3. To estimate adherence to BSCT in three ways: by calculating the number of adherent patients for certain treatment activities; by displaying how the degree of adherence changes over the course of the treatment; and by reporting the proportion of adherent patients according to the threshold for adherence.
4. To explore the possible predictors of adherence to BSCT by examining the relationship between being adherent or nonadherent and 33 person-, smoking-, and health-related characteristics assessed at baseline.

## Methods

### Study Participants

In this study, we used a subset of an RCT on the effectiveness of BSCT versus face-to-face treatment as usual [11]. Patients were referred to the outpatient smoking cessation clinic at the Medical Spectrum Twente hospital (Enschede or The Netherlands) by the treating physicians of the hospital or by the patients’ general practitioners. Inclusion criteria included (1) being at least 18 years old, (2) currently smoking (at least one cigarette a day), (3) having access to email and internet, (4) being able to read and write Dutch. For the adherence analysis, we used the RCT data of the first 75 patients of the BSCT who attended an initial treatment session from May 2015 to December 2016. In line with the Dutch Medical Research Ethics Committee (MREC) guidelines, the study was approved by the accredited MREC Twente (P14-37/NL50944.044.14). Before initiation, the study was registered in the Dutch Trial Registration (NTR5113). All patients had to sign an informed consent form before they were randomized.

### Blended Smoking Cessation Treatment

The BSCT examined in this study is a combination of face-to-face treatment and Web-based sessions blended into one integrated smoking cessation treatment, which is delivered in routine care settings. BSCT consists of 5 face-to-face sessions at the outpatient clinic and 5 Web-based sessions delivered via the Web-based treatment platform. Table 1 shows the order, timing, main features, and mode of delivery of the sessions.

The following are the characteristic features of BSCT:

1. High-intensity treatment: BSCT comprises 10 sessions (20 minutes each, except the first one, which is of 50 minutes); it covers the majority of evidence-based behavior change techniques [44]. It is derived from the Dutch Guideline Tobacco Addiction [45], fulfilling the requirements of the Dutch care module for smoking cessation [46]; the counselors are registered in the Dutch quality register of qualified smoking cessation counselors.
2. Supports three quitting strategies: At the start, patients choose to (1) stop at once, (2) change gradually by increasing the number of daily activities that are performed smoke-free, or (3) decrease smoking at regular intervals (scheduled smoking reduction, eg, 100%->75%, 75%->50%). The chosen quitting strategy does not influence the course of the treatment in general, that is, the order, pace, duration, and intensity are the same for all strategies.
3. A 50-50 balance between face-to-face and Web-based treatments: The focus of the treatment is neither on face-to-face nor on Web-based treatment; in addition, the treatment is constantly alternating and there is interactive use of face-to-face and Web-based treatments.

A detailed description of the treatment can be found in the protocol article of the RCT [11].

**Table 1:** Order, timing, main features, and mode of delivery of blended smoking cessation treatment.

Session	Week	Main features	Mode of delivery
1	1	Goal setting, prompt smoking diary, measure CO^a^	Face-to-face
2	3	Measures for self-control	Web-based
3	5	Dealing with withdrawal	Face-to-face
4	7	Breaking habits	Web-based
5	9	Dealing with triggers	Face-to-face
6	11	Food for thought	Web-based
7	14	Think differently, measure CO	Face-to-face
8	18	Do differently	Web-based
9	22	Action plan, measure CO	Face-to-face
10	26	Closure	Web-based

^a^CO: carbon monoxide.

### Data Collection

#### Patients’ Characteristics and Smoking Status

As part of the RCT, 33 person-, smoking-, and health-related characteristics were assessed with the intake measurement using a Web-based questionnaire. A detailed description of these characteristics is available in the protocol article of the RCT [11]. In addition, both the 3-month and 6-month follow-up measurements of the trial were used to examine the self-reported smoking status.

#### Measuring Adherence to Blended Smoking Cessation Treatment

Two data sources were screened to determine which treatment activities of the patients could be traced after the first treatment session:

1. The patients’ record from the Web-based treatment platform. These records provided, on the one hand, a section where patients and counselors communicated via messages and, on the other hand, a section with therapeutic Web-based tools that were used by the patient. Both sections interact with each other. Here is a typical example of this interaction: The counselor sends a message with instructions to use a therapeutic Web-based tool, such as “goal setting.” With this message, the counselor also unblocks the goal setting Web-based tool and sets a date for executing this task. After receiving this message, the patient uses the unblocked Web-based tool to elaborate goals. What the patient fills in can then be reviewed by the counselor, who also has access to the tool. The counselor then usually responds to what the patient filled in via a message and leads into the following face-to-face session.
2. Patients’ records from the outpatient cessation clinic, which were maintained by the counselors. These records provided additional information about patients’ activities, such as adhering to a stop-date or measurement of CO.

After comparing the treatment manual with the data available in the two data sources, 18 activities of patients were selected to score adherence after the first treatment session. The selection of activities was based on the following three considerations:

1. The activity had to refer directly to a relevant evidence-based behavior change technique [44] (eg, goal setting, action plan) that represented the main feature of the sessions, so that adherence to each of the 10 sessions of the treatment was separately measurable.
2. The activities had to trace both face-to-face and Web-based behaviors of patients (eg, attending face-to-face treatment sessions as in “Think differently [face-to-face]” or completion of predefined Web-based tasks as in “Think differently [Web]”), so that adherence to the constant interaction between face-to-face and Web-based treatments—and by this, the blended nature of BSCT—was covered.
3. The data used had to be objective (eg, receiving a message, unblocking a Web-based tool, filling in a minimal number of data in a Web-based tool) to avoid the limitations of self-reported data [41].

The majority of the selected activities reflected the course of the blended treatment, starting with “Goal setting (face-to-face)” at the end of session 1 and finalizing with “Action plan (Web)” in session 10. Three activities were not session dependent as they had to be executed several times (“Measurement of CO [face-to-face]”) or across several sessions (“smoking diary [days; Web]”; “smoking diary [moments; Web]”). A detailed description of these activities showing how each activity was operationalized to indicate adherence or nonadherence is provided in Table 2.

Based on data sources, for each patient, adherence to each of the 18 activities was assessed and graded adherent or nonadherent by trained research assistants. Finally, for each patient, an adherence score from 0 (adherent to no activity after the first treatment session) to 18 (adherent to all activities) as well as subscores for Web-based versus face-to-face and session-dependent versus session-independent activities were available.

**Table 2:** Activities, operationalization, and patients’ adherence to blended smoking cessation treatment (N=75).

Activity and mode of delivery			Operationalization	Adherent patients, n (%)
**Session-dependent activities**				
	**Session 1: Goal setting**			
		Face-to-face treatment	The patient was introduced to “goal setting” and received a message with the prompt to use the Web-based goal setting tool.	62 (82)
		Web-based treatment	The patient used the Web-based goal setting tool.	44 (58)
	**Session 2: Measures for self-control**			
		Face-to-face treatment	The patient was introduced to “measure for self-control” and received a message with information about measures for self-control.	39 (52)
		Web-based treatment	The patient reacted to the measure for self-control message. (Note: a response was not obligatory; patients could read only without responding)	27 (36)
	**Session 3: Dealing with withdrawal**			
		Face-to-face treatment	The patient was introduced to “dealing with withdrawal” and received a message with information about dealing with withdrawal.	31 (41)
		Web-based treatment	The patient reacted to the dealing with withdrawal message. (Note: a response was not obligatory; patients could read only without responding)	20 (26)
	**Session 5: Dealing with tempters**			
		Web-based treatment	The patient received a message with information about dealing with tempters.	22 (29)
	**Session 6: Food for thought**			
		Face-to-face treatment	The patient was introduced to “food for thought” and received a message with information about food for thought.	17 (23)
		Web-based treatment	The patient reacted to the food for thought message (Note: a response was not obligatory; patients could read only without responding)	8 (11)
	**Session 7: Think differently**			
		Face-to-face treatment	The patient was introduced to “think differently” and received a message with the prompt to use the Web-based think differently tool.	14 (19)
		Web-based treatment	The patient used the Web-based think differently tool.	14 (19)
	**Session 8: Do differently**			
		Face-to-face treatment	The patient was introduced to “do differently” and received a message with the prompt to use the Web-based do differently tool.	12 (16)
		Web-based treatment	The patient used the Web-based do differently tool.	14 (19)
	**Session 9: Action plan**			
		Face-to-face treatment	The patient was introduced to “action plan” and received a message with the prompt to use the Web-based action plan tool.	13 (17)
	**Session 10: Action plan**			
		Web-based treatment	The patient used the Web-based action plan tool.	13 (17)
**Session-independent activities**				
		Measurement of CO^a^ (face-to-face treatment)	The counselor reported at least 2 CO measurements.	34 (45)
		Smoking diary (days; Web)	The patient used the Web-based smoking diary tool registering cigarettes smoked for at least 3 days.	26 (35)
		Smoking diary (moments; Web)	The patient used the Web-based smoking diary tool describing at least 3 moments with an urge to smoke.	31 (41)

^a^CO: carbon monoxide.

### Patients’ Characteristics

Patients’ person-, smoking-, and health-related characteristics were reported as means with SDs for normally distributed continuous variables and as medians with interquartile ranges (IQRs) for not-normally distributed continuous variables. Categorical variables were reported as numbers with corresponding percentages.

#### Dose-Response Relationship Between Adherence and Quitting

To explore the association between the degree of adherence and quitting success, the median number of adherence activities was compared between quitters (based on self-reported smoking status) and smokers at 3 and 6 months after the start of the treatment and tested using Mann-Whitney-U test.

#### Threshold to Detect Adherent Patients

To define a threshold for an adequate degree of adherence, the minimum number of adherence activities of quitters (6 months after the start of the treatment) was examined and displayed as number (%) of activities for BSCT overall and separately for both face-to-face and Web-related activities.

### Adherence to Blended Smoking Cessation Treatment

#### Adherence per Activity

To examine the degree of adherence to each of the BSCT activities, the number (%) of patients fulfilling each activity was examined and displayed separately.

#### Adherence Over the Course of the Treatment

To show changes in adherence over the course of the treatment, the number of patients who were adherent to session-dependent activities was displayed in a bar chart.

#### Adherence Based on the Threshold

The number (%) of patients who were adherent and nonadherent based on the determined threshold to detect adherent patients was cross-tabulated for both the face-to-face and Web-based modes. The number of adherent patients was compared between face-to-face and Web-based treatments and tested using Pearson chi-square test.

### Predictors of Adherence

To identify the predictors of adherence within the 33 person-, smoking-, and health-related patient characteristics, *t* tests or Mann-Whitney-U tests were performed as appropriate for continuous variables; Pearson chi-square or Fisher’s exact test were performed for categorical variables. Variables with a significance *P*<.15 were considered as the candidates for multivariate logistic regression analyses and were entered after checking for multicollinearity. Forward stepwise logistic regression analyses were performed. Variables were entered step for step and were eliminated when the model fit was not significantly increased by adding the variable (based on −2 log likelihood). In case of multicollinearity, the variable with the best model fit was selected for logistic regression analyses.

### Statistical Analysis

All analyses were performed using SPSS version 24.

## Results

### Patients’ Characteristics

Patients’ person-, smoking-, and health-related characteristics are shown in Multimedia Appendix 1.

### Dose-Response Relationship Between Adherence and Quitting

A subsample of patients’ self-reported smoking status at 3 months (n=25) and 6 months (n=17) after the start of the treatment was available to explore the relationship between adherence and quitting. As can be seen by the numbers of activities for adherence tabulated in Table 3, there is a dose-response relationship between adherence to BSCT and self-reported smoking status at 3 and 6 months after the start of the treatment. The median number of activities for adherence is significantly higher among quitters at 6 months after the start of the treatment (*P*=.03).

### Threshold to Detect Adherent Patients

Patients with self-reported abstinence at 6 months after the start of the treatment (n=17) were adherent to at least 61% (11/18) activities of BSCT. Because BSCT is built on a 50%-50% relation for both modes of delivery, a 61% threshold was applied to both modes of delivery to detect adherent patients (ie, patients were defined as adherent if 5 of the 8 face-to-face activities as well as 6 of the 10 Web-based activities were fulfilled).

### Adherence to Blended Smoking Cessation Treatment

#### Adherence per Activity

Table 2 shows the number (%) of adherent patients for each activity. Of all, 17.3% (13/75) patients were adherent to none of the activities, which indicates that these patients did not fully complete the first face-to-face session that closes with the patient being introduced to “goal setting” and receiving a message with the prompt to use the Web-based goal setting tool. None of the patients were adherent to all activities.

#### Adherence per Activity Over the Course of Treatment

**Figure 1:**
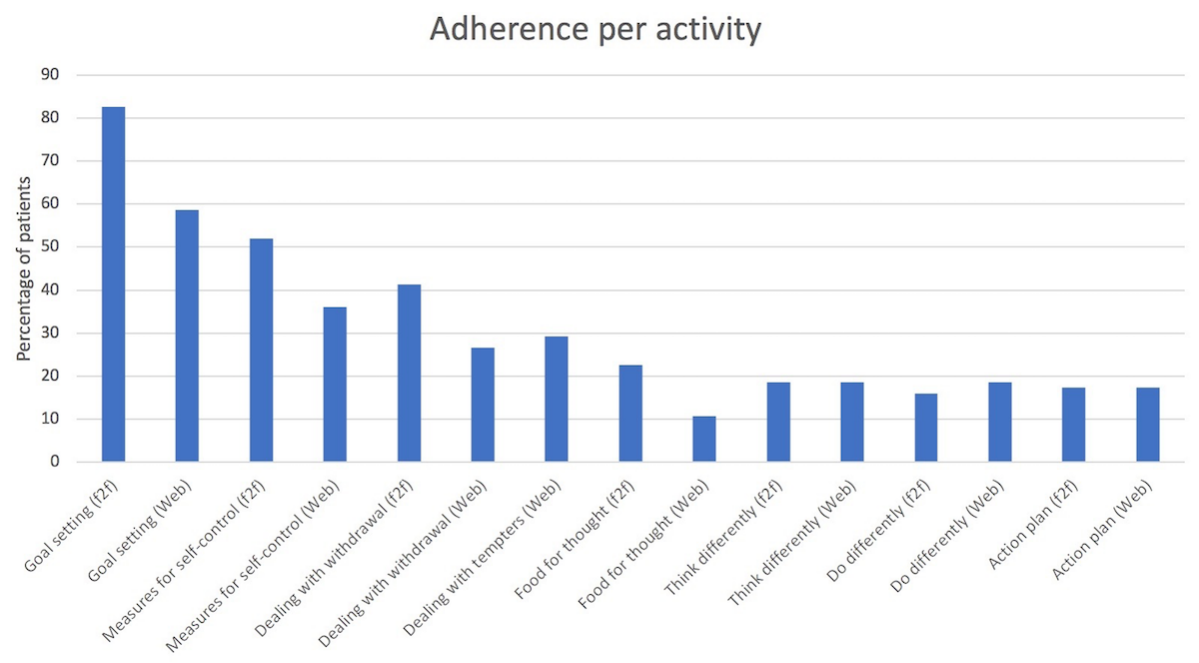
Adherence over the course of the treatment. f2f: face-to-face.

To show the change in adherence over the course of the treatment, the number of adherent patients per session-dependent activity (excluding the 3 session-independent activities) is displayed in a bar chart (Figure 1). The number of adherent patients was highest at the start of the treatment (62/75, 82%, patients adherent to “Goal setting [face-to-face]”). Adherence then decreased to 11% (8/75) for the activity “Food for thought (Web),” which is in the 6^th^treatment session (Table 2), staying at a low level for the rest of the treatment (varying between 12/75, 16%, and 14/75, 19%).

**Table 3:** Adherence to blended smoking cessation treatment and self-reported smoking status at 3 and 6 months after the start of the treatment.

Time point	Median number of adherence activities (IQR^a^)		*P* value
	Quitter	Smoker	
3 months (n=25)	14.5 (9.5-15.8)	9.0 (6.0-14.0)	.08
6 months (n=17)	15.0 (11.8-16.0)	9.0 (6.5-14.5)	.03

^a^IQR: interquartile range.

**Table 4:** Adherence to blended smoking cessation treatment based on the 61% threshold (N=75). Percentages are based on the overall N value.

Face-to-face		Web-based					
	Adherent n (%)		Nonadherent n (%)		Total n (%)	
Adherent		14 (18)		5 (7)		19 (25)
Nonadherent		3 (5)		53 (70)		56 (75)
Total		17 (23)		58 (77)		75 (100)

### Adherence Based on the Threshold

Based on the 61% threshold for both modes of delivery, 18% (14/75) patients were adherent to both modes of delivery and, therefore, to BSCT as a whole (Table 4). Of all, 25% (19/75) patients were adherent to the face-to-face treatment compared with 23% (17/75) adherent to the Web-based treatment (*P*=.70); 70% (53/75) patients were nonadherent to both modes of delivery. Furthermore, 5% (3/75) patients were adherent to the Web-based mode but not to the face-to-face mode, while 7% (5/75) patients were adherent to the face-to-face mode but not to the Web-based mode.

### Predictors of Adherence

The 33 person-, smoking-, and health-related characteristics of the 75 patients, stratified by the 61% adherence or nonadherence threshold, are shown in Multimedia Appendix 1.

The following predictors were univariately associated with adherence (*P*<.15): sex (male = more adherent); marital status (with partner = more adherent); main income (wage or own company = more adherent); social modeling (less smokers in the social environment = more adherent) and use of alcohol (higher alcohol consumption = more adherent); use of other medication (user = more adherent); health-related complaints (as per Maudsley Addiction Profile Health Symptoms Scale [MAP HSS]), smoking-related complaints, and health- and smoking-related complaints (less complaints = more adherent). Due to multicollinearity between health-related complaints (MAP HSS), smoking-related complaints, and health- and smoking-related complaints, only the variable with the best model fit could be included in multivariate regression analysis, which was health- and smoking-related complaints.

Multivariate regression analyses revealed that marital status and social modeling—accounting for 25% of the variance (Nagelkerke R Square)—were independent predictors of whether patients were adherent to BSCT. Patients having a partner had 11 times higher odds of being adherent, although the extremely wide CI indicates considerable uncertainty for this odds ratio (OR=11.3; CI: 1.33-98.99; *P*=.03). For social modeling, graded from 0 (=partner and friends are not smoking) to 8 (=both partner and nearly all friends are smoking), each unit increase was associated with 28% lower odds of being adherent (OR=0.72; CI: 0.55-0.94; *P*=.02).

## Discussion

### Principal Findings

This study is the first to explore adherence to a blended face-to-face and Web-based smoking cessation treatment (BSCT). Based on a substantial group of participants (N=75), the study revealed a rather low adherence rate to BSCT among this sample of outpatients in a regional hospital in the Netherlands. Applying a 61% threshold for adherence to both face-to-face and Web-based modes of delivery, only 18% (14/75) of the participants were classified as adherent. A dose-response relationship was found between the level of adherence and the likelihood of quitting smoking, corroborating the adequacy of the adherence measure developed for this purpose. Furthermore, several baseline characteristics, in particular marital status and social modeling of nonsmoking, were found to be predictive of adherence to BSCT.

So far, the established measures for adherence to blended treatment are still lacking. Therefore, the first aim of this study was to develop an adequate method for measuring adherence to BSCT and to determine whether this method is adequate. Using data from the hospital’s patient records and data logged by patients and counselors on the Web-based treatment platform, a composite score of adherence to 18 distinct treatment activities from both the modes of delivery was calculated. By relying on observed behavior, an objective approach was applied, thus, avoiding bias due to self-report [41]. Adequacy of the adherence measure was confirmed by the observed dose-response relationship between adherence and likelihood of quitting, which is consistent with smoking cessation literature [24,26-28].

A justified threshold for “adequate” and “inadequate” adherence may be useful for clinical purposes [32]. Hence, the second aim of the study was to define an adequate level of adherence to be used as a threshold to detect patients adherent to BSCT. We used a 61% threshold, which is derived from the minimum level of adherence of the patients reporting abstinence. Although this 61% threshold is considerably lower than the commonly applied thresholds of >80% in the previous studies [33,47], it seems realistic if the design of BSCT is taken into consideration. As BSCT is designed as a 10-session, high-intensity treatment, its completion might pose a challenge to the patients of the outpatient clinic. Furthermore, BSCT fosters quitting around month 3 of the treatment while focusing on stabilizing abstinence in the remaining 3 months. Although patients are informed that relapse prevention is pivotal in the later parts of treatment, there seems to be a tipping point at around 60% of the treatment course at which some of the patients who have been successful until then, decide to abandon treatment, thinking themselves “over the hump.” This may also be explained by the bidirectional causality between quitting and adherence: early quitting success predicts adherence [33], while, in turn, adherence predicts (long-term) abstinence [24,26-28].

Because, until now, little has been known about adherence to blended treatment, the third aim of the study was to estimate adherence to BSCT. Not surprisingly, we found a notable decrease in adherence over the course of the treatment. Only a small proportion of patients (12/75, 16%, to 14/75, 19%) was adherent to the last 4 of the 10 BSCT sessions. This is in line with the <40% adherence rates for the later stages of smoking cessation treatment in general [24].

Based on the 61% threshold derived from self-reported abstinence, we found that only 18% (14/75) of the patients were adherent to BSCT. This adherence rate seems to be notably lower than the adherence rates reported in smoking cessation literature, which—while applying even higher thresholds—range from, for example, 50% for Web-based treatment [47] to 70% for face-to-face smoking cessation treatment [34]. Explanations for the low adherence may be negative user experience (eg, due to too demanding or time-consuming features) or lack of persuasive elements [48] in the Web-based treatment.

We also found no significant difference in adherence to Web-based and face-to-face modalities. This is in line with earlier findings, showing that discontinuing the blended treatment is unrelated to the blended nature of the treatment [20].

Since predicting adherence may increase treatment efficacy, the fourth aim of the study was to find the predictors of adherence to BSCT. We found being adherent to be significantly (*P* ≤.05) related to male gender, having a partner, wage or own company as the source of main income, and having a low number of smokers in the social environment. Except for gender [34], we could not confirm predictors earlier reported in the literature, such as age [33,34], internet skills [35,36], attitude toward smoking and motivation to quit [37,38], self-efficacy [38], and nicotine dependency and withdrawal symptoms [34,37]. Furthermore, we found potential predictors of adherence to BSCT (*P* ≥.05-.15) not earlier reported in the context of smoking cessation treatment, namely use of alcohol, use of other medication, and health- and/or smoking-related complaints. Two predictors—having a partner and having a low number of smokers in the social environment—were found to be unique, independent predictors in the multivariate predictor model. Having a partner gave higher odds of being adherent than living alone. Although not earlier reported in the context of smoking cessation, this seems to be consistent with a meta-analysis about marital status and adherence to medical treatment in general, which found 1.27 (CI: 1.12-1.43) times higher odds of adherence in married than in unmarried patients [49]. The most common explanation for this effect of marital status is the social support that a partner may provide to the patient. However, within this study, we did not find a predictive effect of social support, which included the partner as an important other. It should be noted, however, that the measure for social support was specified for smoking cessation and not for adherence. Future research is needed to clarify these inconsistencies. The remarkably high OR of 11 for marital status should be interpreted cautiously because the CI for this ratio was very wide, probably due to limited statistical power in our data. For social modeling, graded from 0 (=partner and friends are not smoking) to 8 (=both partner and nearly all friends are smoking), each unit increase gave 28% lower odds of being adherent. Although not previously reported as a predictor of adherence, social modeling is well known as one of the main determinants of relapse [50-52]. Apparently, patients with more smokers in their environments have a higher probability to relapse; consequently, they drop out of treatment, resulting in lower adherence. This would also be in line with the bidirectional causality between quitting and adherence mentioned above. Looking at social modeling as a relevant predictor of adherence to BSCT, as a clinical implication, the treatment could offer more normative influence (eg, showing videos of peers praising quit attempts of others to patients who report high social modeling of smoking in their environment).

### Limitations

A major limitation of this study is that the results found for BSCT have not been compared with either a face-to-face only or a Web-based only treatment. Therefore, it remains undecided whether the results are specific for the blended nature of the treatment or rather for smoking cessation treatment in general.

In addition, the statistical power in this study—especially for predictor analysis—is rather low. This implies, in particular, that false-negative results may have occurred for predictors with small to medium observed effect sizes. As the purpose of this study is primarily exploratory, the risk of false-negative findings was reduced by also considering marginally significant effects as “potential” predictors. However, caution should be taken here, and replication of these findings in future studies is needed.

Furthermore, the adequacy of the adherence measure can be questioned on three aspects. First, 3 of the 10 Web-based activities, “Measure for self-control,” “Dealing with withdrawal,” and “Food for thought,” involved written messages sent by counselors prompting a response by, for example, asking a question at the end of the message. Only when a patient responded and left a traceable action, the activity was scored as completed. Yet, patients may still have read the received messages without explicitly responding to the counselor. This may have led to an underestimation of adherence because the patient may have been exposed to some components without notable traces. Second, the data from the hospital’s patient records and the Web-based treatment platform partly depend on activities of the counselors because they maintain these patients’ records and also act on the Web-based treatment platform (eg, sending messages to patients, unblocking treatment tools). One can argue, therefore, that adherence measurement is affected by treatment fidelity of counselors. Fidelity of counselors was not evaluated—a common omission in adherence studies [53]. Given that, in this particular case, BSCT was new to the counselors, and their potential unfamiliarity with BSCT may have led to not following the treatment protocol strictly. This may also be increased by therapist drift [54], a common phenomenon in face-to-face cognitive behavioral therapy, which involves a shift from “doing therapies” to “talking therapies.” Third, although striving for objective measures, whether an activity was fulfilled by a patient was doubtful in some cases; for example, the use of the Web-based goal setting tool was questionable if only one word to set a goal (eg, “health”) was sufficient. These cases had to be discussed in the research team, which adds a subjective factor to the measurement. However, this was only the case for a very small number of participants, mainly in the starting phase of data collection.

Finally, the comparability of the observed adherence rates and thresholds across studies is limited due to variety in treatment demands and operationalizations of adherence [32].

### Implication for Future Work

To assess whether the results found in this study are specific for the blended nature of BSCT, the results should be compared with either face-to-face only or Web-based only treatment. Furthermore, future adherence studies should preferably include a measure of fidelity as well, enabling analyses that control for provider-mediated effects on adherence. In addition, from a clinical perspective, the question arises as to how the low adherence rate to BSCT can be increased by, on the one hand, targeting patients to predictive characteristics at baseline or, on the other hand, redesigning BSCT to better accommodate current population characteristics and needs.

## Appendix

Multimedia Appendix 1 Predictors of adherence or nonadherence to blended smoking cessation treatment.

## Abbreviations

BSCT: blended smoking cessation treatment
CO: carbon monoxide
f2f: face-to-face
IQR: interquartile range
MREC: Medical Research Ethics Committee
OR: odds ratio
RCT: randomized controlled trial
