# Supplementary material for: Blended Smoking Cessation Treatment: Exploring Measurement, Levels, and Predictors of Adherence
Source: J Med Internet Res. 2018 Aug 1;20(8):e246. doi: 10.2196/jmir.9969 (PMC6094087; doi:10.2196/jmir.9969)
Supplement: Multimedia Appendix 1 [file jmir_v20i8e246_app1.pdf]

Predictors of adherence or nonadherence to blended smoking cessation treatment.

Predictor			Adherent n=14	Nonadherent n=61	*P* value
**Person related**					
	**Sex**				
		Female (%)	3 (21)	31 (51)	.05[a]
		Male (%)	11 (79)	30 (49)	
	Age in years, median (IQR[b])		55 (38 to 61)	45 (33 to 58)	.17
	**Marital status**				
		With partner (%)	13 (93)	38 (62)	.03[a]
		Alone (%)	1 (7)	23 (38)	
	**Housing situation**				
		With children (%)	6 (43)	26 (43)	.99
		Without children (%)	8 (57)	35 (58)	
	**Education**				
		VET[c] or higher (%)	7 (50)	36 (59)	.54
		Lower than VET (%)	7 (50)	25 (41)	
	**Main income**				
		Wage or own company (%)	11 (79)	30 (49)	.05[a]
		Income support (%)	3 (21)	31 (51)	
	**Main day activity**				
		Paid work (%)	10 (71)	31 (51)	.16
		Other (%)	4 (29)	30 (49)	
	Internet skills[d], median (IQR)		36.5 (35.8 to 40.5)	38.0 (34.0 to 41.0)	.98
**Smoking related**					
	**Reason to start the treatment**				
		Intrinsic (%)	11 (79)	40 (66)	.53
		Extrinsic (%)	3 (21)	21 (34)	
	Nicotine dependency (Fagerstroem[e]), median (IQR)		5 (4 to 7)	6 (4 to 7)	.53
	Negative attitude toward quitting[f], median (IQR)		−5.0 (−6.0 to −3.5)	−6 (−3.5 to −9.0)	.33
	Positive attitude toward quitting[g], median (IQR)		9.5 (8.0 to 10.3)	10 (8.0 to 11.5)	.69
	Self-efficacy[h], median (IQR)		0 (−3.3 to 5.3)	−1 (−5.0 to 3.0)	.34
	Readiness to quit[i], median (IQR)		2.0 (1.0 to 3.0)	2.0 (1.0 to 3.0)	.88
	**Earlier quit attempts**				
		Yes (%)	11 (79)	51 (84)	.70
		No (%)	3 (21)	10 (16)	
	Social support[j], median (IQR)		4.0 (3.0 to 4.3)	4.0 (3.0 to 4.0)	.57
	Social modelling[k], median (IQR)		2.0 (0.0 to 4.3)	4.0 (1.0 to 6.0)	.05[a]
	Use of alcohol[l], median (IQR)		3 (1 to 4)	2 (1 to 3)	.08[a]

	**Use of (recreational) drugs**				
		Yes (%)	0 (0)	8 (13)	.34
		No (%)	14 (100)	53 (87)	
**Health related**					
	**Use of medication in general**				
		Yes (%)	8 (57)	36 (59)	.90
		No (%)	6 (43)	25 (41)	
	**Use of medication for addiction treatment**				
		Yes (%)	0 (0)	0 (0)	—[m]
		No (%)	14 (100)	61 (100)	
	**Use of medication for psychiatric treatment**				
		Yes (%)	3 (21)	9 (15)	.69
		No (%)	11 (79)	52 (85)	
	**Use of medication for physical treatment**				
		Yes (%)	4 (29)	29 (48)	.20
		No (%)	10 (71)	32 (52)	
	**Use of other medication**				
		Yes (%)	4 (29)	6 (10)	.08[a]
		No (%)	10 (71)	55 (90)	
	Health-related complaints (MAP HSS)[n] Median (IQR)		9.5 (6.8 to 13.0)	11.0 (7.0 to 19.0)	.08[a]
	Smoking-related complaints[o] Median (IQR)		17.0 (9.5 to 22.3)	21.0 (15.5 to 27.5)	.13[a]
	Health- and smoking-related complaints[p], median (IQR)		28.5 (13.8 to 35.3)	37.0 (23.5 to 47.5)	.08[a]
	Depression[q], median (IQR)		4.0 (1.5 to 13.0)	4.0 (0.0 to 10.0)	.75
	Anxiety[q], median (IQR)		3.0 (2.0 to 6.0)	4.0 (2.0 to 9.0)	.52
	Stress[q], median (IQR)		5.0 (1.5 to 13.5)	8.0 (4.0 to 15.0)	.34
	DASS[r], median (IQR)		14.0 (4.0 to 32.5)	20.0 (9.0 to 31.0)	.49
	Euroqol-5D[s], median (IQR)		0.80 (0.73 to 1.0)	0.80 (0.69 to 1.0)	.27
	Euroqol-5D VAS[t], median (IQR)		70 (63 to 80)	70 (59 to 80)	.86

[a]Variable entered in the multivariate logistic regression model based on *P* value ≤ 0.15.

[b]IQR: interquartile range.

[c]VET: vocational education and training.

[d]Internet skills: range, 10-60; higher number indicates better skills.

[e]Fagerstroem: higher numbers indicate higher nicotine dependency.

[f]Negative attitude toward quitting: lower numbers indicate a more negative attitude toward quitting smoking.

[g]Positive attitude toward quitting: higher numbers indicate a more positive attitude toward quitting smoking.

[h]Self-efficacy: higher numbers indicate higher self-efficacy related to smoking cessation.

[i]Readiness to quit: higher numbers indicate higher readiness to quit.

[j]Social support: higher numbers indicate more social support in smoking cessation.

[k]Social modeling: higher numbers indicate more smokers in the social environment.
[l]Use of alcohol: higher numbers indicate higher alcohol consumption.
[m]No *P* value computed because variable is a constant.
[n]MAP HSS: Maudsley Addiction Profile Health Symptoms Scale (higher numbers indicate poorer health status).
[o]Smoking related complaints: higher numbers indicate more smoking-related complaints.
[p]Health- and smoking-related complaints: higher numbers indicate poorer health status and more smoking-related complaints.
[q]Depression, Anxiety, and Stress (higher number indicates a higher level of depression, anxiety, and stress).
[r]DASS: sum score of Depression, Anxiety, and Stress (higher numbers indicate more negative emotional status).
[s]Euroqol-5D: societal-based quantification of patients' health status (higher numbers indicate better health status).
[t]Euroqol-5D VAS: visual analogue scale for quality of life (higher numbers indicate better state of health).
